# Adeno-associated virus serotype 9 structural heterogeneity and stability characterized by charge detection mass spectrometry

**DOI:** 10.1016/j.omtm.2025.101608

**Published:** 2025-10-06

**Authors:** Zachary M. Miller, Li F. Lin, David V. Schaffer, Evan R. Williams

**Affiliations:** 1 Department of Chemistry, University of California, Berkeley, Berkeley, CA 94720-1460, USA; 2 Department of Chemical and Biomolecular Engineering, University of California, Berkeley, Berkeley, CA 94720-1460, USA; 3 California Institute for Quantitative Biosciences, University of California, Berkeley, Berkeley, CA 94720-1460, USA; 4 Department of Bioengineering, University of California, Berkeley, Berkeley, CA 94720-1460, USA; 5 Department of Molecular and Cell Biology, University of California, Berkeley, Berkeley, CA 94720-1460, USA; 6 Helen Wills Neuroscience Institute, University of California, Berkeley, Berkeley, CA 94720-1460, USA; 7 Innovative Genomics Institute (IGI), University of California, Berkeley, Berkeley, CA 94720-1460, USA

**Keywords:** adeno-associated virus, AAV, gene therapy, gene delivery, charge detection mass spectrometry, CDMS, mass spectrometry, heterogeneity, native MS, viral capsid structures

## Abstract

The variability in structure and stability of adeno-associated virus serotype 9 (AAV9) in response to changes in buffer composition, pH, and temperature was investigated using charge detection mass spectrometry (CDMS). AAV9 virus-like particles (VLPs) consisting of only viral protein (VP) 3 and wild-type AAV9 capsids (i.e., capsids containing varying stoichiometries of VP1, VP2, and VP3) showed differences in structure, indicating that these different VP stoichiometries and compositions may contribute substantially to conformational heterogeneity. Significant differences in AAV9 structure and stability were observed in ammonium acetate (AA) vs. phosphate buffered saline (PBS) solutions under some conditions. At 37°C under acidic conditions, AAV capsids fell apart in AA, whereas in PBS, capsids underwent structural compaction. Subsequent nuclease binding experiments indicated that partially extruded DNA was the likely origin of this structural compaction that occurred under different physical and chemical conditions. Results from one freeze-thaw cycle indicated that the capsids degraded by a similar mechanism to that in acidified solution. The structural complexity revealed by CDMS highlights the advantages of this biophysical characterization method in providing, for the first time, a holistic insight into the potential heterogeneous conformational transitions of AAV9 during purification, storage, and the natural infection process.

## Graphical abstract

**Figure:**
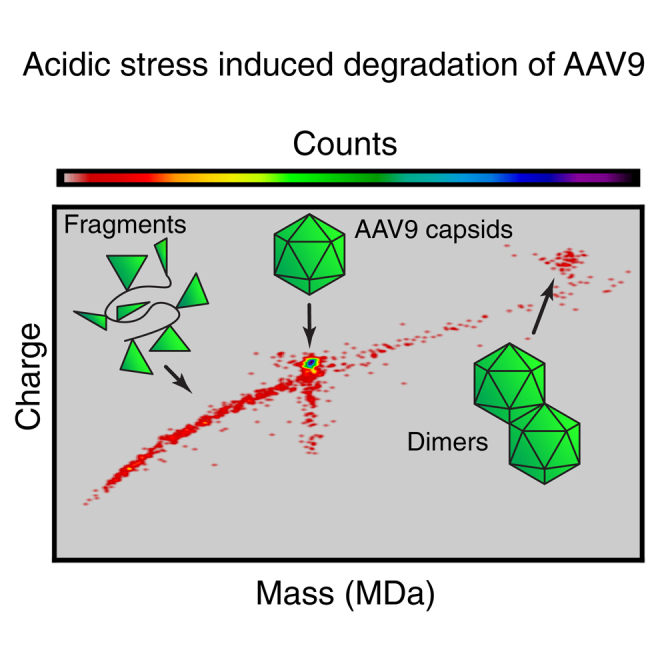

## Teaser

Williams and colleagues used charge detection mass spectrometry to investigate the structures and stability of adeno-associated virus, serotype 9 (AAV9). AAV9 had multiple conformers that depended both on buffer composition and temperature. Partial extrusion of DNA at physiological pH with structural compaction provides insights into natural infection processes.

## Introduction

Adeno-associated viruses (AAVs) are single-stranded, non-enveloped DNA viruses of the *Parvoviridae* family and the *Dependovirus* genus. In recent years, these viruses have received elevated interest due to their promise as effective gene therapy vectors.^1^^,^^2^^,^^3^ These viral capsids contain 60 copies total of three viral proteins (VPs 1, 2, and 3) at an average stoichiometry of ∼1:1:10 but with some variability among virions.^4^^,^^5^^,^^6^ The virions are relatively small (24–26 nm)^5^ and encapsulate a ∼4.7 kilobase viral genome flanked by inverted terminal repeats (ITRs). AAV’s genome is categorized into replication (*rep*) and structural (*cap*) genes, which encode replication and capsid-related proteins, respectively.^7^

AAVs offer numerous advantages as DNA delivery vehicles, including the efficient transduction of non-dividing cells, a lack of pathogenicity, an episomal genome with minimal host genome integration or genotoxicity, and a recombinant genome containing only the desired DNA cargo flanked by the ITRs. As of December 2024, recombinant AAVs have been used in over 136 gene therapy-related clinical trials, as well as seven FDA-approved gene therapies targeting the retina, central nervous system, liver, and muscle. While these successes highlight the promising potential of AAV therapies, adverse side effects^8^^,^^9^ have emerged in the clinic, particularly at high AAV doses. This motivates further investigations to understand the interplay between AAV and immune response to enable engineering of more efficient and safe AAV-based gene delivery.

DNA sensing receptors act as innate immune sentinels that surveil the presence of aberrant oligonucleotides, such as viral genomes. Notably, AAV transduction has been reported to activate both Toll-like receptor 9 (TLR 9) and cGAS-STING pathways.^10^^,^^11^^,^^12^ TLR9-mediated activation of immune responses has been reported in *ex vivo* transduction of plasmacytoid dendritic cells with AAV8, resulting in the upregulation of pro-inflammatory cytokines, as well as *in vivo* dosing of C57BL/6 mice, where TLR9 signaling is strongly correlated with a cytotoxic T cell response.^11^^,^^12^ However, contradictory results have been reported by Kajaste-Rudnitski and coworkers, which suggest that TLR9 activation plays an insignificant role toward immune activation in hiPSC-derived neurons and astrocytes.^10^ Instead, a cytosolic DNA sensing pathway—cGAS-STING— is shown to be necessary to elicit pro-inflammatory responses following AAV administration.^10^

The activation of both TLR9 and cGAS-STING pathways requires premature exposure of AAV viral genome—specifically, extrusion of the genetic cargo from the capsid following endocytosis but prior to nuclear localization. One possible cause is endosomal acidification, a key process that triggers capsid conformational rearrangements to facilitate viral endosomal escape and that compromises the structural integrity of a subset of AAV virions and trigger genome release. The structures of AAV8, AAV9, and SAAV capsids have been resolved under different pH conditions using cryo-electron microscopy (cryo-EM), revealing distinct structural rearrangements within the 5-fold channel of these capsids.^13^ Specifically, channel opening was observed in SAAV, whereas a tightening of the channel was noted in AAV9 at lower pH. Other characterization methods, such as differential scanning fluorimetry (DSF) and immunoblotting, were also utilized to investigate pH-dependent AAV stability^14^ and indicate that AAVs are most thermally stable at pH ∼6.0–5.5. Both DSF and immunoblotting are effective approaches for evaluating bulk biophysical properties, but these techniques may overlook finer details that are hidden in the heterogeneity that is intrinsic to these particles. Similarly, cryo-EM relies on averaging thousands of images to reconstruct particle structures, which may mask low abundance structures or stoichiometries with distinct properties that coexist in solution.^15^^,^^16^^,^^17^^,^^18^ Notably, to date, no studies have directly demonstrated premature exposure of AAV genomic cargo under conditions mimicking the endocytic environment. This gap in characterization further confounds our understanding of how AAV genome-mediated innate immune activation is initiated.

Charge detection mass spectrometry (CDMS) is a rapidly developing technique that enables swift analysis of high molecular weight particles.^19^^,^^20^^,^^21^ Electrospray ionization is used to transfer analytes from solution into the gas phase^22^ where single particle mass measurements are made in an electrostatic linear ion trap.^23^^,^^24^^,^^25^^,^^26^ CDMS is different from conventional mass spectrometry measurements in that the masses of individual ions can be obtained through simultaneous measurements of *m/z* and charge (*z*), thereby circumventing spectral congestion that can obfuscate interpretation of conventional MS spectra of high mass, heterogeneous analytes.^27^ Thousands of individual ion measurements are then compiled into particle mass distribution histograms. CDMS has been used to investigate properties of AAVs, such as capsid stoichiometries, empty vs. full capsid ratio, the integrity of packaged DNA, thermal stability, and antibody binding.^19^^,^^28^^,^^29^^,^^30^^,^^31^^,^^32^^,^^33^^,^^34^

Volatile buffers, such as ammonium acetate (AA) or ammonium bicarbonate, are used in native MS to provide high solution ionic strength without the extensive salt adduction and cluster formation that occur with conventional biochemical buffers that have high concentrations of nonvolatile salts, such as phosphate buffered saline (PBS). Salt adduction and salt clusters can lead to broad and overlapping peaks in mass spectra, which can confound the extraction of useful mass information.^35^ Nano-electrospray ionization (nESI) emitters with submicron diameter tips enable characterization of biomolecular complexes from non-volatile salt solutions by generating droplets that are sufficiently small that each one has the probability of only containing one or no analytes and relatively few salt ions.^35^^,^^36^ This advance makes it possible to measure mass spectra of biological samples in buffers that contain non-volatile salts, including sodium chloride, which are commonly used in biochemistry. The identity of different salts in solution is well known to affect the stability of proteins and other biomolecules, and the Hofmeister series ranks ions based on their propensity to stabilize or destabilize secondary and tertiary structure.^37^ Effects of Hofmeister salts on protein structure have been widely investigated.^38^^,^^39^^,^^40^ Previous mass spectrometry results show differences in protein stability and structure in biochemically relevant buffers, such as Tris and 1xPBS, compared to AA, which is used in nearly all native MS experiments.^40^ To date, all experiments aimed at characterization of AAV biophysical properties with CDMS have been carried out using AA buffers with neutral pH.^28^^,^^31^^,^^33^

In this study, the effects of pH on AAV9 structure and stability are investigated. Evidence for extensive conformational heterogeneity that has not been observed previously, and a new degradation pathway are presented along with results in both AA and PBS buffers that show pronounced differences in both AAV stability and conformation. Collectively, these results provide evidence for possible premature AAV genome exposure during endocytosis and reveal the importance of buffer systems and the advantages of CDMS for gaining insights into protein and virion stability and structure.

## Results

### Effects of different buffers on AAV9 structure

**Figure 1:**
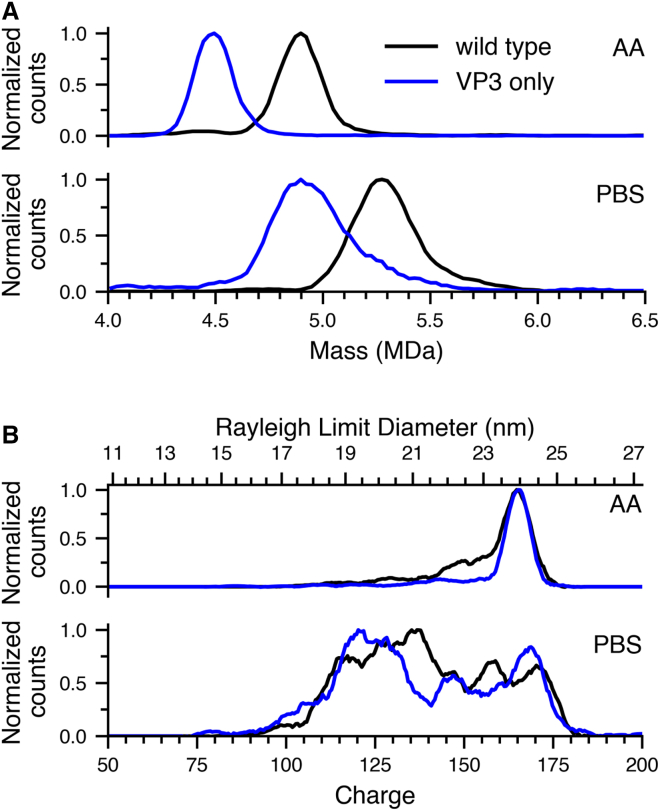
Structural differences between AAV9 and VP3-only VLPs and dependence on buffer (A) Mass histograms of AAV9 (black line) and VP3 only VLPs (blue line) in 500 mM ammonium acetate (AA; top) and 1xPBS (with extra 200 mM NaCl) (PBS; bottom); (B) Charge histograms of AAV9 (black line) and VP3 only VLPs (blue line) in AA (top) and PBS (bottom). The top axis in (B) is the particle diameter determined from the comparable charge corresponding to the Rayleigh limit computed for an aqueous spherical nanodrop using Equation 1.

CDMS mass histograms of CMV-GFP AAV9 and CAG-GFP virus-like particles (VLPs) consisting of just VP3 in 500 mM AA and 1xPBS (with an additional 200 mM NaCl added to the buffer solution) solutions are shown in Figure 1A. Both buffer conditions were selected to maintain elevated ionic strength to minimize AAV aggregation.^41^ The centroid masses of AAV9 and VLPs measured from the AA solution are 4.93 MDa and 4.51 MDa, respectively. These masses differ both because of the different capsid protein composition and the different DNA cargo mass (1.05 MDa for CMV-GFP and 0.84 MDa for CAG-GFP). The calculated mass for CMV-GFP AAV9 is 4.91 MDa, based on the molecular weights of VP1 (83.5 kDa), VP2 (68.6 kDa), VP3 (62.0 kDa) and assuming a 1:1:10 (VP1:VP2:VP3) stoichiometry. In comparison, the CAG-GFP VP3-only capsid is calculated to have a mass of 4.54 MDa, assuming a stoichiometry of 60 VP3 subunits only.^34^ Thus, the measured and calculated masses are in excellent agreement, consistent with many other CDMS measurements of AAV masses from AA solutions.^29^^,^^42^^,^^43^ The centroid masses of these same two AAVs when measured from the PBS solutions are 5.28 MDa and 4.90 MDa, respectively, or 0.38 and 0.42 MDa higher than the corresponding masses in AA. This mass difference results from extensive adduction of salts and phosphate ions onto the intact AAV particles due to the high concentrations of these nonvolatile salts in the PBS buffer (337 mM NaCl, 2.7 mM KCl, 10 mM Na_2_HPO_4_, and 1.8 mM KH_2_PO_4_).^35^^,^^36^

Adduction of nonvolatile salts, such as NaCl onto proteins and DNA ions commonly occurs even after buffer exchange into nonvolatile buffers, such as AA. For example, salt adduction onto ions of large protein complexes (∼365 kDa–800 kDa) in AA can lead to measured masses that are 0.3%–1.2% higher than the calculated masses.^44^ In prior work, the masses of AAV9 with DNA cargo formed from 1xPBS and from an elution buffer containing ∼300 mM of nonvolatile solutes were higher by ∼120 and ∼520 kDa, respectively, compared to these ion formed from AA solution.^45^ The mass shift to 5.28 MDa from the PBS solution containing high concentrations of nonvolatile salts (Figure 1A) is consistent with the mass shifts observed in this earlier work. Importantly, the mass of the DNA cargo can still be obtained from the mass difference between AAVs with and without cargo (when present) even with extensive salt adduction because both forms of the AAVs adduct similarly.^45^ Extensive adduction also leads to broader peaks in the mass histogram that reflects the heterogeneity of the adducted species that are formed.^45^

The charge state of an ion in native MS is related to its surface area, shape, and surface properties.^28^^,^^46^^,^^47^^,^^48^ CMDS measurements of antibody and other protein aggregates have shown that aggregates with the same mass but different conformations in solution can be readily differentiated based on differences in charging.^47^^,^^48^ The charge histograms for the mass data shown in Figure 1A are shown in Figure 1B. From AA, the most abundant charge state for both AAV9 and VLP was ∼165*e*, indicating that these ions are comparable in physical size. For AAV9, there was a lower abundance shoulder centered at ∼150*e* and a tail that extended down to ∼110*e* with <10% abundance. The VLP charge histogram was similar to that of AAV9 but without the lower charge shoulder. This suggests that there is more conformational heterogeneity due to the presence of different stoichiometries of three different proteins in AAV9 compared to capsids formed from just one protein. In PBS, the charge-state distributions of both AAV9 and VP3-only VLPs were significantly different from those in AA. These charge states extended to lower values (95*e* and 90*e*, respectively), and these lower charge states were significantly more abundant than those formed from AA. The AAV9 charge histogram in PBS appeared to be comprised of seven distributions with least squares fitted centroids at 171, 159, 147, 137, 128, 117, 102*e*, whereas the VLP charge histogram appeared to be comprised of only five distributions with fitted centroids at 167, 148, 131, and 120, 102*e*, (Figures S1A and S1B, respectively). These broad charge-state distributions indicate that numerous capsid conformations must coexist in solution, and the more even population abundance over the range of charge states of AAV9 compared to VP3-only VLPs show that VP1 and VP2 must contribute to structural heterogeneity of AAV9 capsids.

The differences between charge histograms measured from AA and PBS solutions for both AAV9 and VLPs indicate that the structures of the complexes differ in these buffers. Many prior CDMS measurements on multiple AAV serotypes obtained from AA solutions indicate that full capsids have charge histograms that resemble those shown in Figure 1B (top) where just a dominant single charge-state distribution is observed along with a tail down to lower charge.^28^^,^^31^ Distinctly lower charge-state distributions have been observed for empty AAV8 capsids in AA, even though a single high charge-state distribution was observed for filled capsids present in the same sample.^31^ The lower charge-state distribution was attributed to capsid compaction during the nESI process, and it was hypothesized that the DNA cargo within filled particles prevents compaction. Results from both cryo-TEM and gas phase electrophoretic mobility measurements also indicate that no measurable structural change occurs in packaged AAVs in AA over this range of pH.^49^

Although the charge histograms of both AAV9 and VLPs from PBS had a peak at 170*e*, the majority of ions had much lower charge. This indicates that a significant fraction of these cargo-containing AAV capsids had more compact structures. The distribution of highest charge states in AA and PBS correspond to diameters of ∼24.0 nm and 24.5 nm, respectively, based on their charging relative to that calculated for a water droplet of comparable size using Equation 1.^50^^,^^51^ This diameter is within the range of ∼25 nm that has been previously reported for filled AAV particles.^5^ The lowest charge-state distributions for both AAV9 and VLPs from PBS approached ∼19.5 nm on the Rayleigh limit diameter axis (top *x* axis in Figure 1B), corresponding to ∼5 nm of compaction. The maximum extent of charging of AAV9 and VLPs ions from PBS (Figure 1B) was slightly higher than that from AA. Emission of Na^+^ from the droplets has been proposed as a mechanism that can reduce macromolecular charging in some cases.^52^ Formation of higher charge states when sodium ions are present in solution rule out this mechanism for reducing the extent of charge here since emission of Na^+^ cannot both reduce and increase charge. The appearance of multiple distributions in the charge histograms of both AAV9 and VLP ions formed from PBS provides evidence that these distributions are not formed by emission of Na^+^ but must be a result of multiple structures that are stable in this buffer. This observation is particularly interesting as these AAV9 populations retain viral genomes as indicated by their mass yet still undergo structural compaction. A plausible explanation is that the presence of different cations in the PBS vs. AA buffer causes viral genome conformational heterogeneity, allowing for capsid structural rearrangements.^53^ Compaction of viral particles at low pH is not unprecedented. Similar compaction effects have been reported from cryo-EM data of Cowpea chlorotic mottle virus under acidic conditions.^54^ However, to our knowledge this is the first report of a range of more compact structures for packaged AAVs.

Differences between charge histograms of AAV9 and VLPs in both buffers provide additional evidence that the structural families of capsids consisting of VP1, VP2, and VP3 are different from VP3-only capsids, though these differences appear to be relatively minor. CDMS measurements of MS2 capsids from both AA and sodium phosphate (measured masses of ∼3.42 MDa and 3.67 MDa, respectively) showed one dominant charge-state distribution in both buffers, consistent with a singular structure.^52^ In contrast, the multiple charge-state distributions observed for these AAV ions formed from PBS indicate that multiple different structures coexist in solution. These results highlight the importance of forming AAV ions from solutions containing appropriate concentrations of biochemically relevant salts.

### pH stability in ammonium acetate vs. phosphate buffer

AAVs exploit the endosomal-lysosomal network for intracellular trafficking to the nucleus.^13^ During this process, AAV capsids experience a gradual pH decrease from physiological pH (∼7.4) to an acidic pH as low as ∼4.0.^13^ Previous cryoEM reconstructions of acidified, empty AAV9 indicated that pore-like structures (generally referred to as the 5-fold channel) along the outside of the capsid appear to close as pH decreases, though these measurements were conducted only on capsids without DNA cargo, which might impact capsid stability.^53^

**Figure 2:**
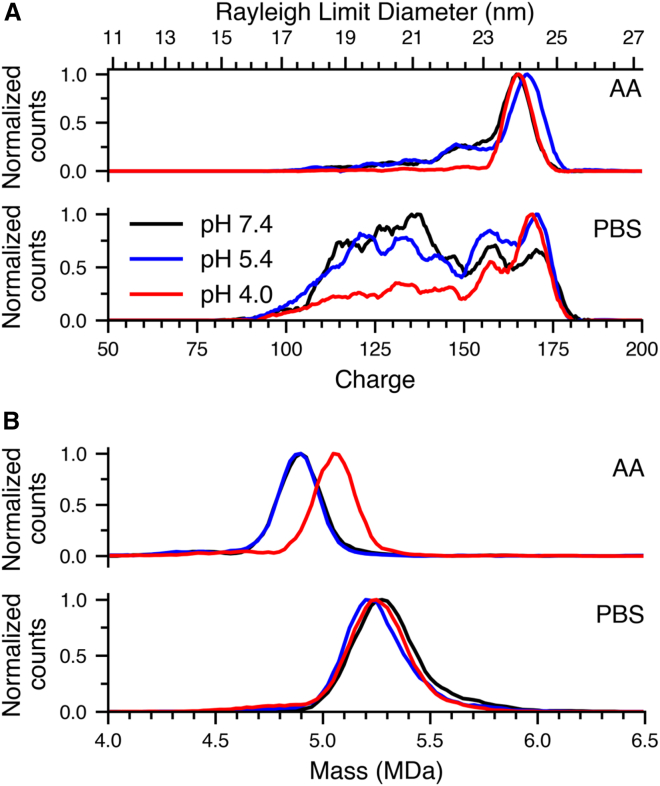
Effects of pH and buffer composition on AAV9 structure at 4°C (A) Charge histograms of AAV9 incubated at pH 7.4 (black line), 5.4 (blue line), and 4.0 (red line) at 4°C in AA (top) and PBS (bottom); (B) Mass histograms of AAV9 incubated at pH 7.4 (black line), 5.4 (blue line), and 4.0 (red line) at 4°C in AA (top) and PBS (bottom).

AAV stability can be very sensitive to the storage buffer, where the temperature of thermal denaturation was found to vary in AAV3 by up to ∼20°C in Tris vs. citrate-phosphate solutions.^55^ To further investigate the effects of buffer composition on AAV stability, AAV9 containing a CMV-GFP genome was buffer exchanged into either AA or PBS at pH 7.4, 5.4, or 4.0, and incubated at 4°C for 30 min. CDMS data were then acquired from these six solutions. The charge histograms (Figure 2A) from AA are comprised of a single dominant distribution at the same charge for each pH, indicating that the majority of AAV9 capsids in AA have similar structures regardless of pH. Charge histograms for both pH 7.4 and 5.4 in AA buffer also had a low abundance shoulder toward lower charge states of similar abundance, whereas the lack of these states in pH 4.0 AA buffer suggests that the less compacted, higher charge states are more stable at this lower pH.

In striking contrast, the charge histograms of AAVs in PBS were more complex and showed significantly more structural heterogeneity (Figure 2A, bottom). The charge histogram from PBS pH 7.4 solution indicated that compacted structures were in higher abundance along with the presence of multiple structural conformations. As was the case for AA, the charge histogram from PBS at pH 5.4 was similar to that at pH 7.4, whereas there was a significant shift to more highly charged, less compact structures in PBS at pH 4.0. A similar effect was observed in AA (Figure 2A, top), though the data in PBS showed that a much larger fraction of the population underwent this structure shift at this low pH. Overall, charge histograms from both buffers showed similar trends, where lower charge states had higher relative abundances for solutions closer to neutral pH, though the relative abundances of charge states in PBS appeared to be far more sensitive to solution pH compared to AA.

Mass histograms in Figure 2B showed additional differences in the behavior of AAV9 capsids in different pH AA or PBS buffers. Centroid masses of AAV9 in PBS at pH 7.4, 5.4, and 4.0 were 5.28, 5.23, and 5.26 MDa, respectively. Small differences in centroid mass in PBS were consistent with varying extents of adduction because of minor variations in the initial droplet sizes produced by nESI with different emitters. Similarly, the centroids of the mass distribution of AAV9 at pH 7.4 and 5.4 from AA were both 4.90 MDa. However, the centroid mass was 5.07 MDa in AA at pH 4.0. This ∼0.16 MDa mass shift is reproducible and corresponds to a 3.3% increase in mass compared to that measured from pH 7.4 or pH 5.4 solutions. This pH-dependent mass shift in AA is unexpected. The concentration of ammonium is essentially unchanged (pK_a_ = 9.25), but acetate converts to acetic acid at low pH (pK_a_ = 4.76). Ammonium is often used in native mass spectrometry because it can proton transfer to the macromolecular complex and readily leave as ammonia. Adduction of acetate or acetic acid is not commonly observed, even at pH 3.3.^56^ This mass shift only occurs at pH 4.0, whereas pH 5.4 and 7.4 data do not show any detectable mass increase. There is no evidence of capsid degradation under these conditions, so the added mass must be either water or buffer molecules as opposed to non-specific adduction of non-volatile capsid or DNA fragments.

At pH 4.0, the water or buffer molecules could be trapped in the interior of the AAV9 capsids or between capsid proteins as is typical with protein crystals.^57^ Cryo-EM reconstructions of AAV9 VLPs showed that the 5-fold pores located on the exterior of AAV9 capsids are closed under acidic conditions.^13^ The masses of pure aqueous nanodrops in this size range have been measured using CDMS,^58^ and aqueous nanodrops with an average mass of 5.1 MDa evaporated approximately 319 kDa of water molecules per second. Water and ion weakly bound to AAV ions may likewise be lost prior to or during measurements in the electrostatic ion trap.^58^ However, no detectable mass loss from AAV ions occurred during these mass measurements (Figure S2), indicating that any residual water and ions are restricted from evaporating because they are contained inside the capsid or closely bound between capsid proteins and do not readily leave due to the structural rearrangements (e.g., tightening of the 5-fold channels) in acidified AA buffer. In contrast, results from PBS do not show this same trend of increasing mass with decreasing pH observed from AA solutions. This may be a result of their already high mass due to an abundance of adducted non-volatile salt ions that are more tightly bound and do not leave as readily.

### Effect of temperature on stability

**Figure 3:**
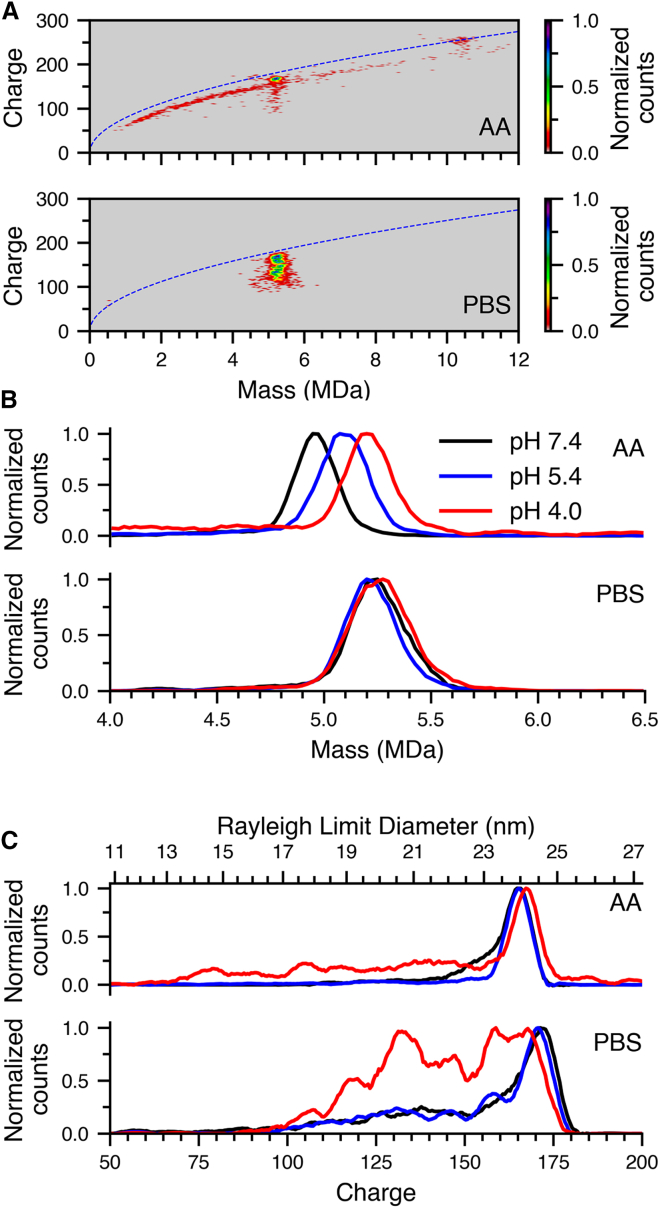
Effects of pH and buffer composition on AAV9 structure and stability at 37°C (A) Two-dimensional mass/charge histograms of AAV9 in pH 4.0 AA (top) and PBS (PBS) incubated at 37°C. The blue dashed line corresponds to the Rayleigh limit for a spherical water droplet as a function of mass. (B) Mass histograms of AAV9 incubated at pH 7.4 (black line), 5.4 (blue line), and 4.0 (red line) at 37°C in AA (top) and PBS (bottom); (C) Charge histograms of AAV9 incubated at pH 7.4 (black line), 5.4 (blue line) and 4.0 (red line) at 37°C in AA (top) and PBS (bottom).

Samples of AAV9 capsid were heated to physiological temperature (37°C) for 30 min, then cooled to room temperature, and mass and charge histograms were obtained from both AA and PBS solutions at pH 7.4, 5.4, and 4.0. This difference between heated vs. control, unheated samples is most clearly apparent in the two-dimensional mass/charge histograms of AAV9 incubated at 37°C at pH 4 in AA. In AA, there were intact, full capsids, but there was also a broad distribution of ions from 1 MDa to 12 MDa (Figure 3A, top). This distribution intersects with the distribution of AAV9 ions at ∼150*e*, which coincides with the shoulder adjacent to the main charge-state distribution measured at pH 7.4 and 5.4 AA in the 4°C data (Figure 2A). There is no signal for empty capsid at the expected mass (∼3.9 MDa or up to ∼4.1 MDa with adducts) and charge (∼160*e*),^45^ indicating that DNA is not ejected from an intact capsid. Instead, the charging of these ions as a function of mass trends with the Rayleigh limit (Figure 3A blue dashed line). The masses and charges of these ions appear as a continuum that extends both below and above the expected mass of the empty AAV9 capsid and exhibit significantly lower charge than that of empty capsid. This indicates that acidic conditions in AA at elevated temperatures destabilize the capsid itself, leading to solution-phase fragmentation of the capsid through the loss of constituent proteins. Masses above the intact full capsid also appear, consistent with aggregation of some constituent proteins lost from some capsids onto intact AAV9. Dimers of AAV9 also appear alongside the degradation and aggregation products at lower mass. The dimer species is charged at the Rayleigh limit, consistent with a dimer of two intact AAV9 capsids that can charge slightly more than a spherical structure at that same mass. In striking contrast, the two-dimensional mass/charge histograms of AAV9 incubated at 37°C at pH 4 in PBS shows only intact AAV9 with no loss of constituent proteins or aggregates thereof, nor are there any dimers formed.

There were also significant differences in the pH dependence of the mass data obtained from the two buffers (Figure 3B). As was the case at 4°C, the mass of AAV9 shifts significantly higher at pH 4.0 in AA, but there was also an increase in measured mass in the pH 5.4, 37°C data. That is, the centroid masses at pH 7.4, 5.4, and 4.0 for AAV9 in AA were 4.98, 5.12, and 5.22 MDa, respectively. These shifts correspond to increases in centroid mass relative to pH 7.4 at 4°C (Figure 2B) of 0.07, 0.22, and 0.32 MDa, respectively. In contrast, no such shift with pH occurred in PBS (Figure 3B, bottom), where the centroids at pH 7.4, 5.4, and 4.0 were 5.25, 5.24, and 5.27 MDa, respectively. The charge state data from AA at 37°C (Figure 3C, top) showed predominantly a single high charge-state distribution centered at ∼167*e* at the three different solution pH values, indicative of a single structure or family of closely related structures. Thus, the increasing mass with decreasing pH for data acquired from AA solutions is not consistent with significant structural reorganization of the capsid but rather, it is indicative of additional water and ions. The absence of detectable water loss (Figure S3) indicates that the excess mass must be due to trapped water and salts in the interior of the capsid. The charge histogram at pH 4.0 (Figure 3C, top, red data) had a tail down to ∼95*e* that is due to the smaller fragments formed by loss of the constituent proteins from the intact capsid (Figure 3A, top).

In PBS, charge histograms obtained at pH 7.4 and 5.4 were very similar (Figure 3C, bottom, black and blue data) with a main distribution centered at ∼171*e* and a tail down to ∼95*e*, whereas the pH 4.0 data appeared to be composed of six charge-state distributions centered at ∼166, ∼159, ∼145, ∼133, ∼120, and ∼108*e* (Figure 3C, bottom, red data). This suggests a compaction in structure at low pH. An interesting result was that the pH effect on charging was different in the 4°C and the 37°C data in both buffers. This is especially apparent in PBS where decreasing pH led to more abundant high charge states indicating less compact structures are more stable at 4°C (Figure 2A, bottom, red data), whereas decreasing the pH led to lower charge states indicating a preferential compaction in structure for the 37°C data (Figure 3C, bottom, red data). This effect is less apparent in AA where the capsids substantially dissociate, aggregate, and/or form dimers at low pH. These data show that the favored structures of these AAV capsids are highly dependent on temperature, pH, and buffer compositions.

Replicate acidification experiments with PBS solutions at both 4°C and 37°C were performed with an AAV9 sample from the same lot number and supplier but acquired at different times and analyzed seven weeks apart (Figure S4). The shapes of the charge-state envelopes of AAV9 at 37°C are reproducible with minor changes in some peak positions (Figure 3C bottom compared to Figure S4B). The charge-state envelopes at 4°C show a similar trend in shifting to higher charge at lower pH (Figure 2A bottom compared to Figure S4A) but the change at pH 4 is more subtle. The presence of multiple conformers that can readily interconvert with relatively small changes in temperature indicates that the energetic differences between these conformers are small. Consequently, the minor changes in sample preparation or measurement conditions, i.e., time since thaw, room temperature, etc.,^59^ can readily shift the populations of these structures. The better reproducibility of the data at 37°C is consistent with the population more readily overcoming interconversion barriers at the higher temperature that lead to an equilibrium and thus more reproducible population of conformers upon cooling to lower temperature.

### Mechanism of degradation

**Figure 4:**
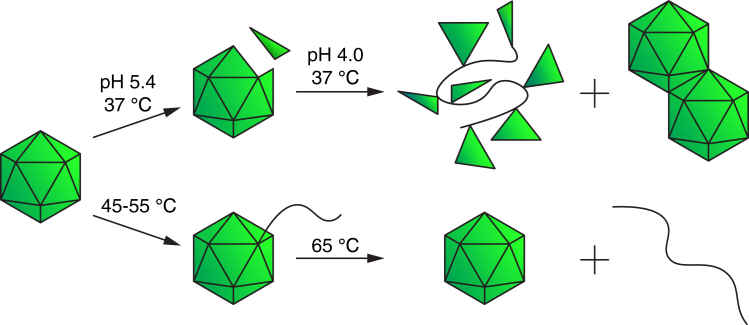
Schematic representation of AAV9 degradation mechanism deduced from these experiments (top pathway) compared to schematic representation of an alternate degradation mechanism reported from high solution temperature measurements (bottom pathway)^28^^,^^60^

The distribution of masses below that of the intact AAV in the pH 4.0 AA solution (Figure 3A) indicates that degradation of AAV9 occurs by loss of individual constituent proteins. The absence of empty AAV9 indicates that protein loss occurs while the DNA is retained by the remaining capsid structure (Figure 4, top pathway). This leads to a wide range of fragment masses, adduction of now free capsid proteins to intact virus, and dimer formation. The trend in charging of the degraded capsids intersects the distribution of charging of the intact capsid at ∼150*e* and not at the most abundant charge state (165*e*). This indicates that degradation occurs through a specific more compact conformer of the AAV9 that is not the most abundant form under a wide range of solution conditions (Figure 3A, top). This mechanism is very different than purely heat-induced degradation at a much higher temperature, as reported by multiple groups: Jarrold and coworkers using CDMS,^28^ Faivre-Moskalenko and coworkers using AFM,^60^ and McKenna and coworkers using cryo-EM,^61^ that resulted in extrusion of an elongated form of DNA and ultimate loss of the DNA leaving intact empty capsids (Figure 4, bottom pathway). Interestingly, heat stress is also used as a surrogate for acidification to detect and study externalization of VP1 and VP2 unique domains.^62^^,^^63^ However, our study shows that degradation of AAVs at low pH is different from that at high temperature, highlighting the dynamic nature of the AAV capsid and its sensitivity to varying chemical environments.^62^^,^^63^ Our results also show that AAV9 capsids are destabilized in AA buffers, making it important to investigate the structures and stabilities of these types of viruses in solutions that contains ionic salts that better mimic biological environments.

### Nuclease binding to AAV9 capsids

**Figure 5:**
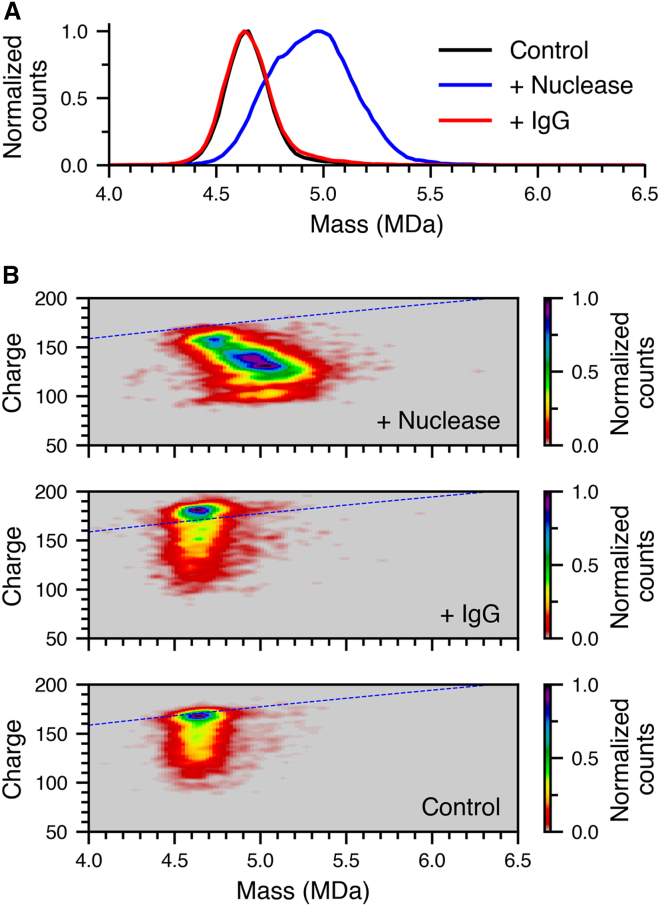
Nuclease binding to AAV9 (A) Mass histogram of AAV9, AAV9 + nuclease, and AAV9 + IgG in black, blue and red, respectively. (B) Two-dimensional mass/charge histogram of AAV9 + nuclease (top), AAV9 + IgG (middle), and AAV9 (bottom). The blue dashed corresponds to the Rayleigh limit for a spherical water droplet as a function of mass.

A possible origin of the different charge states corresponding to different structures in solution is partial DNA extrusion from the capsid structures. To test this hypothesis, nuclease digestion experiments were performed. Nuclease is an enzyme that binds to DNA and cleaves the phosphodiester bond that links two nucleic acid bases. To determine if any DNA cargo is sufficiently exposed to solvent to enable nuclease binding and DNA cleavage, Benzonase was added to AAV9 packaged with CAG-GFP at a ratio of 0.5 U per 1 × 10^11^ AAV9 in 20 μL of 500 mM AA buffer at pH 7.4. In the absence of nuclease, the CDMS data showed the expected mass and charge-state distributions for this sample (Figures 5A, black data and 5B, bottom), similar to Figure 1A despite the slightly different DNA cargo. In the presence of nuclease, there was a significant increase in mass and the width of the peak (Figures 5A, blue data and 5B, top), indicative of nuclease binding to the capsids. The absence of any lower mass species suggests that substantial cleavage of extruded DNA did not occur to a measurable extent, consistent with low activity of this nuclease enzyme in solutions with high ionic strength or that lack divalent cations, such as Mg^2+^.^64^^,^^65^ To confirm that the nuclease enzyme activity is inhibited in the AA solutions used here, its activity was determined by digestion of linearized plasmid DNA in both 1xPBS (137 mM NaCl) and 500 mM AA buffers with and without 2 mM MgCl_*2*_ (Figure S5). The enzyme is active in 1xPBS +2 mM MgCl_*2*_, but is significantly inhibited in 500 mM AA + 2 mM MgCl_*2*_ (Figure S5), consistent with inhibition at elevated ionic strength.

The difference in the centroid of this peak compared to AAV9 only control corresponded to 15 bound nuclease enzymes (∼34 kDa each). A two-dimensional mass/charge histogram in the region of the AAV9-nuclease complexes is shown in Figure 5B, top. There is a clear trend showing that higher mass, i.e., more bound nuclease enzymes, correspond to lower charging or more compact structures. The low-mass end of the distribution begins at the same mass (∼4.6 MDa) and charge (∼160*e*) as the AAV9 only spectrum (Figure 5B, bottom), indicating that the unbound complexes likely maintain their capsid structure in the absence of nuclease. With an increasing number of bound nucleases, the charge state linearly decreases ending at ∼5.1 MDa and ∼120*e*. A similar trend in lower charging was reported for AAV8 upon antibody binding and was attributed to a substantial structural change in the capsid.^30^ As a control, the addition of donkey anti-mouse IgG to a final concentration of 150 nM (corresponding to molar ratio of 50 IgG: 1AAV9) does not affect the peak centroid or width (Figures 5A, red data and 5B, middle). The complete absence of higher mass ions with addition of IgG further supports the interpretation that nuclease attachment is specific binding as opposed to non-specific aggregation induced by the electrospray process.

The correlation between nuclease binding and the formation of lower charge states (Figure 5B, top) provides some insights into the origin of the lower charge states that are also formed in the absence of nuclease (Figure 5B, middle, bottom). Without nuclease, the lower charge states are at the same mass as the higher charge states. However, in the presence of nuclease, lower charge states shift to higher masses, indicating that the lower charge states correspond to capsids that have partially extruded DNA cargo. As more DNA is extruded, the capsid may collapse, reducing overall surface area and ultimately leading to a lower overall charge state. Additionally, the extruded, negatively charged DNA may also interact with the positively charged residues on the capsid surface, thereby further decreasing the net charging by electrospray. Notably, there is also a low-abundance distribution of ions centered at ∼100*e* and a mass of ∼5.0 MDa that deviates from the general trend of decreasing charging with increasing nuclease binding. The distribution may be attributed to the nominal nuclease activity of Benzonase-mediated DNA cleavage for capsids that have the largest extent of extrusion and structural compaction. The observation may also indicate that steric hinderance could influence the extent of the nuclease-mediated DNA cleavage, particularly in capsids still retaining the majority of their DNA cargo.

### Freeze-thaw degradation

**Figure 6:**
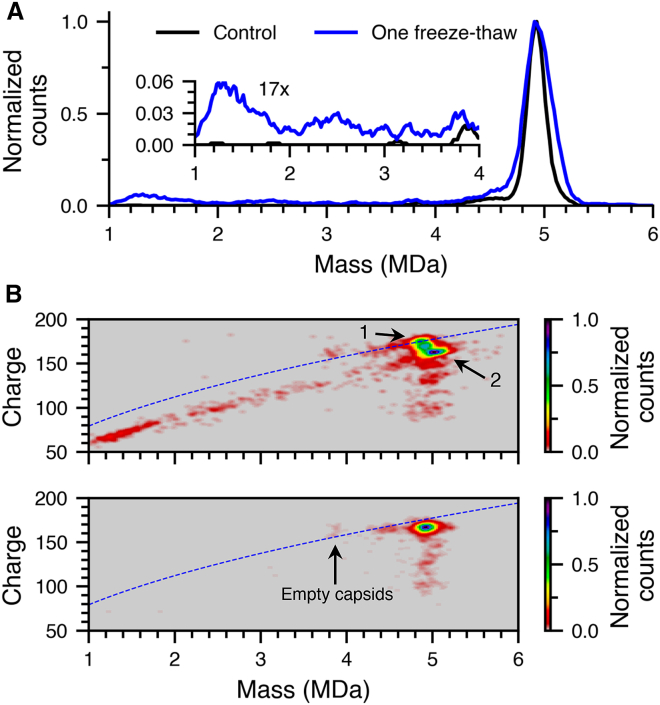
Effects of a freeze-thaw cycle on AAV9 stability (A) Mass histograms of AAV9 before (black) and after one freeze-thaw cycle (blue). The inset is a 17× expansion from 1 to 4 MDa. (B) 2D mass/charge histogram of AAV9 after one freeze-thaw cycle (top) and before one freeze-thaw cycle (bottom). A lower mass, but higher charge distribution is annotated as “1”, and a higher mass, lower charge distribution is annotated as “2” in the top panel. The blue dashed corresponds to the Rayleigh limit for a spherical water droplet as a function of mass.

Many biological complexes can degrade upon freezing and subsequent thawing, a concern for cold storage of vector. To investigate how a freeze-thaw cycle affects AAV9, the same sample of AAV9 packaged with CAG-GFP (at pH 7.4 in 500 mM AA) that was used to investigate nuclease binding was frozen at −80°C for 1 h and thawed at room temperature for 1 h. A mass histogram before (black data) and after one freeze-thaw cycle (blue data) is shown in Figure 6A. The intact AAV9 is centered at the same mass, but the distribution is broader after a single freeze-thaw cycle. Degradation of the capsid occurs after the freeze-thaw cycle (Figure 6) resulting in fragments that are similar to those produced at pH 4.0 in AA at 37°C (Figure 3A), pointing to a similar degradation mechanism. The degradation products are ∼16% of the total ion abundance indicating that substantial degradation has occurred, likely due to both the AA buffer and the absence of any cryoprotectants, such as D-sorbitol, that was in solutions of CMV-GFP AAV9 samples that were frozen (data in Figures 1, 2, and 3).

There are also some distinct differences from the pH stress data (Figure 3A). The AAV9 had two closely spaced distributions: one is lower in mass but higher in charge than the distribution before freeze-thaw (Figure 6B, top, annotated as “1”), and the other extends higher in mass and slightly lower charge (Figure 6B, top, annotated as “2”) than that of the sample before freeze-thaw (Figure 6B, bottom). As was the case with the low pH, 37°C data, this higher mass distribution is likely due to constituent capsid proteins from the degraded sample adducting onto intact AAV9, and this adduction appears to depend on the conformation of the capsid. There was a low abundance (<2%) of empty AAV9 in the initial sample prior to freeze-thaw stress (Figure 6B, bottom). The abundance of empty AAV9 after the freeze-thaw cycle remained below 2% of intact AAV9, indicating that DNA was not ejected from intact AAV9.

## Discussion

The structures and stabilities of AAV9 depend on solution temperatures, pH, and buffer conditions. The AAV structures and stabilities can differ significantly in AA solutions that are commonly used in native mass spectrometry compared to those in phosphate buffer more commonly used in biology. Conformational heterogeneity was indicated by a broad range of charge states with multiple peaks under some conditions, which suggest several different conformers or conformational families of AAVs can coexist in solution. The information from charge-state distributions is analogous to that obtained from much more widely used ion mobility MS from which information about solution structures for a wide range of biomolecules and biomolecular complexes has been inferred.^66^ Multiple conformers of protein aggregates have been identified based on resolved peaks in the charge-state distributions of a given aggregate with CDMS.^47^^,^^48^ While general information, such as a compaction or expansion of structures, can be deduced directly from charge-state distributions, more detailed structural information will require modeling and/or additional experimental data from complimentary structural methods.

The structural heterogeneity identified here may also explain some temperature-dependent results from affinity-based purification of AAV9 using AAVX chromatography, where ∼50% capsid was lost at ∼4°C, but less than 5% was lost ∼24°C.^67^ This effect can be rationalized by our CDMS results where AAV9 capsid had a significantly greater population of compacted conformers in PBS at 4°C than at 37°C. These compacted conformations likely render the surface epitopes less accessible for antibody binding, therefore leading to capsid loss in flow-through.

A capsid consisting of only VP3 protein showed virtually no structural heterogeneity in AA buffer, indicating that different compositions of the VP1-3 proteins may lead to increased conformational heterogeneity. Incubation of AAV9 at 37°C for 30 min in AA at pH 4 led to extensive disassembly of the capsid structure through a specific conformer that was not the most abundant conformer of the intact capsid. This indicates that the different conformers that can be identified based on their extent of charging can have different stabilities. Disassembly occurred by loss of constituent proteins while the remaining assembly that included the DNA remained compact. Both adduction of proteins to and dimerization of intact capsids also occurred. Furthermore, a similar dissociation process occurred upon a single free-thaw cycle. In contrast to thermal stress at higher temperatures where loss of elongated DNA cargo from intact capsids was reported, our results showed a different mechanism in which the DNA appeared to remain in the core of a disintegrating proteinaceous shell.

Results from a phosphate buffer under these same conditions were remarkably different: a significant fraction of the capsids collapsed in size but remained intact. Nuclease digestion experiments in AA buffer revealed that these lower-charge, more compact but intact capsids partially extruded DNA, rendering them accessible to nuclease binding and potential cleavage. These results indicate that the compaction in the conformation of the capsids occurs concurrently with partial extrusion of DNA. Most importantly, this study provides the first direct evidence of AAV capsids prematurely extruding its genome cargo under acidification, bridging the gap between *ex vivo* characterization and *in vivo* observation of immune stimulation. These results contribute to the growing body of research on AAV capsid heterogeneity and highlight the advantages of CDMS for investigating heterogeneous structures, conformational transitions, and physical properties of AAVs in solutions that better mimic biological environments, including potential therapeutic applications.

## Materials and methods

### AAV packaging and production

AAV9-CMV-GFP vectors were obtained from the vector core at the University of North Carolina at Chapel Hill and stored at −80°C. AAV9-CAG-GFP vector used in nuclease and freeze thaw experiments were produced and purified using the same protocol as VP3 only particles described here to minimize extreme pH and temperature variations.

AAV9 and VP3 only particles with CAG-GFP transgene were packaged via triple transfection of HEK293 cells. In brief, 6 μg of pCAG-GFP plasmid, 12 μg of pHelper plasmid, and 10 μg of pRep2Cap9, or pRep2Cap9VP3 plasmid, with GCC mutations substituting VP1 and VP2 start codons, were mixed with polyethyleneimine (PEI) and transfected into ∼90% confluent HEK293 cells. Cell pellets were harvested 96 h post-transfection and lysed with AAV lysis buffer (150 mM Tris, 500 mM NaCl, 4 mM MgCl_2_, 0.5% TX-100, pH 8.5) for 5 h. Lysate was then diluted with ultrapure water to a final concentration of 200 mM NaCl. To degrade and remove the genomic and non-encapsidated DNA, lysate was treated with Benzonase (100U/mL, Invitrogen) at 37°C for five hours. The lysate was then clarified with two sequential, 10-min spins at 3,000 rcf and 10,000 rcf.

### AAV purification

Clarified lysate was loaded onto iodixanol gradients and centrifuged at 42,000 rpm for 2 h using a VTI 65.2 rotor. Fractions at the 40%–54% interface were extracted and dialyzed into storage buffer (1xPBS with 350 mM total NaCl, 0.01% Pluronic F-68). The AAV-containing solution was then loaded into ÄKTA Go system equipped with a HiPrep 16/60 Sephacryl S-300 HR column, pre-equilibrated with the storage buffer. The flow rate was set to 0.2 mL/min and 1 mL fractions were collected. AAV containing fractions were pooled and dry CsCl was added to adjust the final density to 1.40 g/mL, followed by 48-h centrifugation using a SWTi 41 rotor. Fractions from CsCl gradients were collected with a 21 gauge needle. The fraction with the highest AAV concentration, as determined by qPCR, was dialyzed into storage buffer and kept at 4°C.

### AAV acidification

AAV9-CMV-GFP vectors stored at −80°C were thawed at 4°C prior to use. Solutions with concentrations of 1 × 10^13^ viral genomes/mL were buffer exchanged into solutions at various pH (500 mM AA or 1xPBS with 350 mM NaCl and 0.01% Pluronic F-68, adjusted to pH 7.4, 5.4, and 4.0) using Amicon Ultra filters with 5 min of centrifugation at 5000 rcf. In addition to salts that comprise the AA or PBS buffers, ∼60 μM of CsCl remains in the samples from CsCl gradient purification after dialysis and buffer exchange. AAV samples were then incubated at either 4°C or 37°C for 30 min in a pre-equilibrated thermocycler (BioRad). Samples were then filtered with a 0.22 μm spin filter (Costar Spin-X, Corning) immediately before analysis.

### Nuclease treatment

AAV vectors were first buffer exchanged as described above, followed by the addition of 0.5U of Benzonase Nuclease (Cat# 70664, MilliporeSigma) with 2 mM MgCl_2_. As a negative control, donkey anti-mouse IgG antibody conjugated with Alexa Flour 488 (Cat#A21202, ThermoFisher) were incubated with AAV9 at 50:1 molar ratio. The mixtures were incubated at 37°C for 30 min. Samples were filtered with 0.22 μm spin filter prior to analysis.

### Charge detection mass spectrometry

Experiments were performed with a custom-built CDMS instrument.^21^ Ions are generated by nanoelectrospray using borosilicate emitters with tip diameters of ∼800 nm. Ions pass through an ion funnel into a region with three quadrupoles where they are trapped and subsequently introduced into an electrostatic cone-trap that has a cylindrical detector electrode. Ions are trapped for 500 ms, and their induced signals are amplified by a charge-sensitive preamplifier (Amptek A250 CoolFET). Time-domain data are sampled at 1 MHz and are analyzed in real time using a program written in C++ that performs rectangularly apodized (unapodized) short-time Fourier transforms (STFT), picks and fits peaks up to the fourth harmonic in each STFT segment and uses frequencies and amplitudes of the fits to determine individual ion masses and charges.^43^^,^^68^ An STFT with a segment length of 25 ms and step size of 5 ms was used in all experiments. Individual ion mass and charge histograms were fit to Gaussian distributions using a non-linear least squares algorithm to obtain centroid values. To obtain information about the diameters of AAV9 structures in solution, charging of aqueous nanodrops of various sizes based on the Rayleigh limit for a spherical water droplet was determined. This limit indicates the maximum charge state that a stable spherical droplet can have based upon density and surface tension.^69^ Most globular proteins and protein complexes charge near this Rayleigh limit.^21^^,^^46^ The diameter of a spherical water droplet charged at the Rayleigh limit, *d*_*R*_, is given in Equation 1, where *z*_*R*_ is the Rayleigh limit charge, *ε*_*0*_ is the permittivity of the surrounding media, and *γ* is the surface tension of water.

(Equation 1) $$d_{R}={2\cdot \left(\frac{z_{R}}{8\pi }\right)}^{\frac{2}{3}}\cdot {\left(\varepsilon_{0}\gamma \right)}^{-\frac{1}{3}}$$

## Data and code availability

Data will be made available upon request.

## Acknowledgments

This work was supported by the 10.13039/100000002National Institutes of Health (grant number 5R01GM139338) for development and construction of the CDMS instrument, development of data analysis methods used in this work, and writing, as well as 10.13039/100000002NIH grant numbers 1U01MH130700 and 1R01NS126397 for AAV production, purification, sample preparation, analysis, and writing. Z.M.M. acknowledges support from an ACS Graduate Research Fellowship sponsored by 10.13039/100004312Eli Lilly and Company, and Agilent University Relations: ABP Program Grant ID #5116.

## Author contributions

Z.M.M. and L.F.L. performed the experiments. Z.M.M., L.F.L., D.V.S., and E.R.W. conceived of the study, analyzed the data, and wrote the manuscript.

## Declaration of interests

The authors declare the following competing interests: E.R.W. and Z.M.M. are inventors on patents related to CDMS technology. E.R.W. has a position as Chemist Faculty at the Lawrence Berkeley National Laboratory. The work described in this manuscript is unrelated to this position. D.V.S. is an inventor on patents related to AAV capsid engineering and is a member of the editorial board for *Molecular Therapy*.

## Supplemental information

Document S1. Figures S1–S5

Document S2. Article plus supplemental information
