# Supplementary material for: Adeno-associated virus serotype 9 structural heterogeneity and stability characterized by charge detection mass spectrometry
Source: Mol Ther Methods Clin Dev. 2025 Oct 6;33(4):101608. doi: 10.1016/j.omtm.2025.101608 (PMC12641567; doi:10.1016/j.omtm.2025.101608)
Supplement: Document S1. Figures S1–S5 [file mmc1.pdf]

OMTM, Volume 33

Supplemental information

Adeno-associated virus serotype 9 structural heterogeneity and stability characterized by charge detection mass spectrometry

Zachary M. Miller, Li F. Lin, David V. Schaffer, and Evan R. Williams

Gaussian Fitting. Individual ion charge histograms were fit to the sum of multiple Gaussian distributions using non-linear least squares. Fitting was performed using Python version 3.12.6 and the "curve_fit" function in SciPy version 1.14.1.

Frequency Shift Analysis. STFTs of time domain signals showing individual AAV9 ion frequencies were computed as described previously with rectangular apodization (i.e., unapodized).[1] The time evolution of AAV9 ions were traced using a custom built program written in the C++ programming language. To compute an average change in frequency vs. time, AAV9 traces were fit to a line using linear least squares with a slope in units of Hz / 5 ms. Most AAV9 ion signal frequencies are appear constant with time indicating that the ions did not change measurably in mass (indicated by upward slope in frequency)[2, 3] or charge (indicated by a prompt decrease in frequency) during the trapping period.[2, 4]

Benzonase activity assay. The enzymatic activity of Benzonase nuclease was assessed by digestion of linearized plasmid DNA. The pSSGFP plasmid was first linearized using *AflII-HF* restriction enzyme (New England Biolabs), followed by purification with a PCR purification kit (Monarch, New England Biolabs) according to the manufacturer's protocol. A total of 200 ng of linearized plasmid DNA was incubated in either 1xPBS or 500 mM ammonium acetate buffer, each supplemented with 2mM $MgCl_2$ and 0.5U Benzonase nuclease. Samples were incubated at 37 °C for 30 minutes. The extent of DNA digestion was visualized by electrophoresis on a 0.8% (w/v) agarose gel stained with SYBR™ Safe DNA gel stain (Thermo Fisher Scientific).

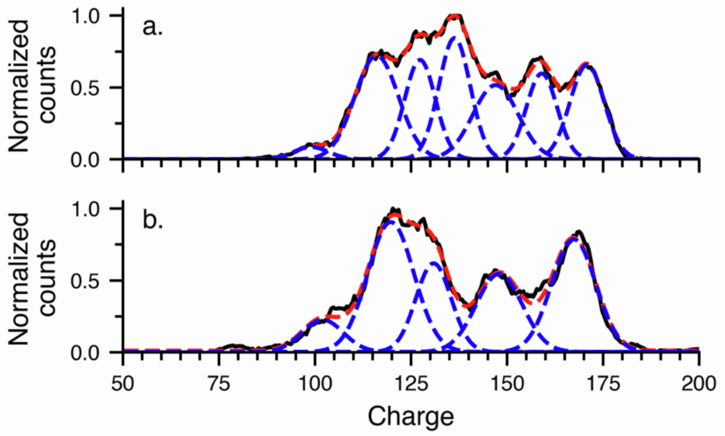

Figure S1. Charge histograms of AAV9 (a) and VP3 only virus-like particles (b) fit to the sum of seven and five gaussian distributions, respectively. Experimental data are plotted as solid black lines, individual Gaussian components are plotted as blue dashed lines, and the sum of Gaussian components are plotted as red dashed lines.

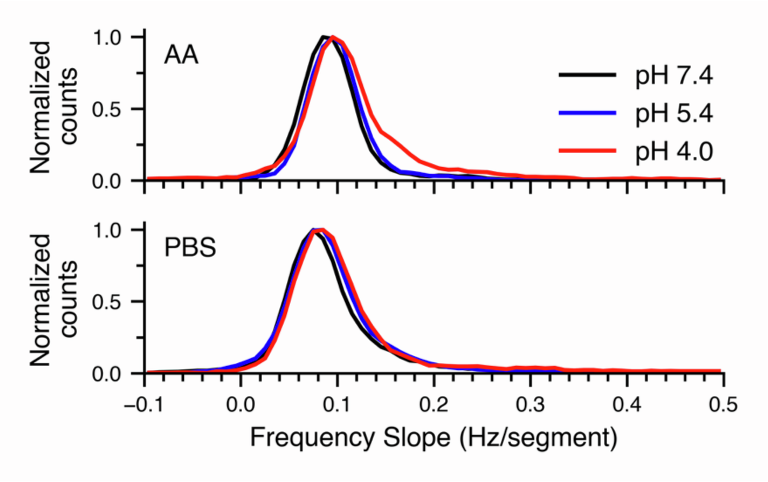

Figure S2. Individual ion average frequency shift histograms for AAV9 incubated in 4 °C AA (top) and PBS (bottom). pH 7.4, pH 5.4 and pH 4.0 are plotted black, blue and red, respectively.

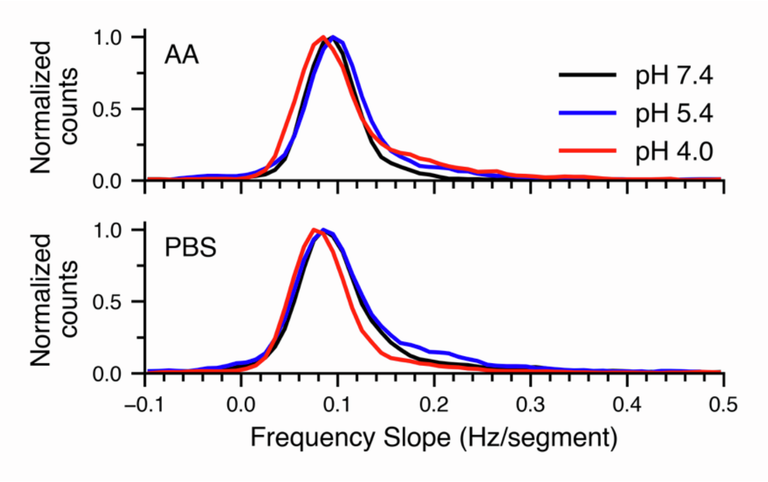

Figure S3. Individual ion average frequency shift histograms for AAV9 incubated in 37 °C AA (top) and PBS (bottom). pH 7.4, pH 5.4 and pH 4.0 are plotted black, blue and red, respectively.

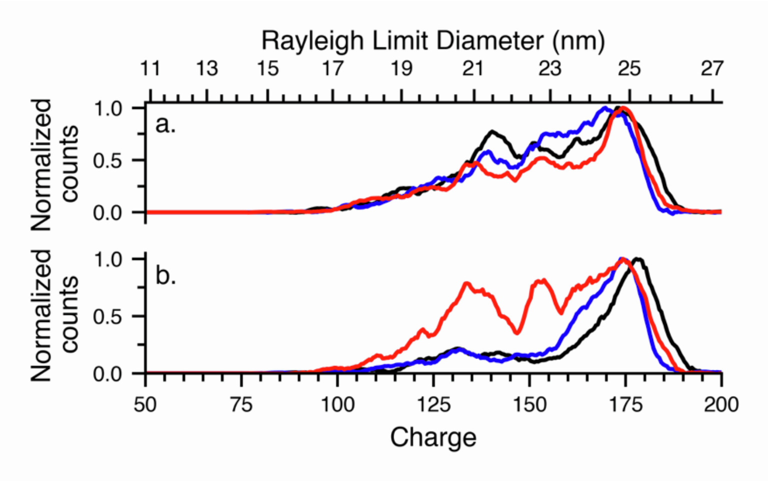

Figure S4. Replicate measurements of AAV9 in pH 7.4 (black data), pH 5.4 (blue data) and pH 4.0 (red data) that were incubated at (a) 4 °C and (b) 37 °C.

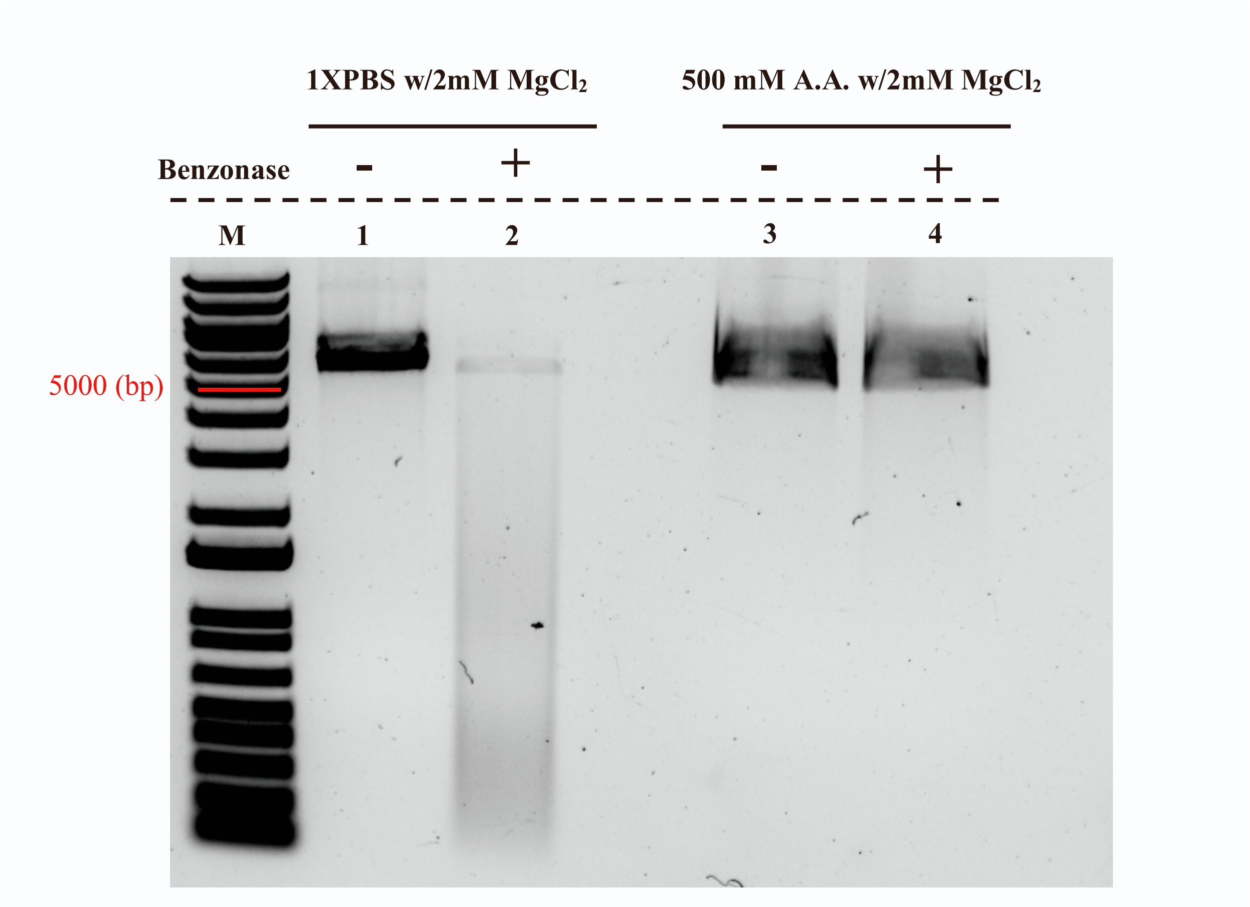

Figure S5. Agarose gel electrophoresis of linear plasmid incubated with Benzonase in different buffer conditions. Lane 1 and 2: Incubation in 1xPBS with 2mM $MgCl_2$; lane 3 and 4: incubation in 500 mM AA with 2mM $MgCl_2$. M: Invitrogen 1 kb plus ladder.

References

(1) Miller, Z. M.; Harper, C. C.; Lee, H.; Bischoff, A. J.; Francis, M. B.; Schaffer, D. V.; Williams, E. R. Apodization Specific Fitting for Improved Resolution, Charge Measurement, and Data Analysis Speed in Charge Detection Mass Spectrometry. *J. Am. Soc. Mass Spectrom.* **2022**, *33* (11), 2129-2137. DOI: 10.1021/jasms.2c00213.

(2) Harper, C. C.; Brauer, D. D.; Francis, M. B.; Williams, E. R. Direct observation of ion emission from charged aqueous nanodrops: effects on gaseous macromolecular charging. *Chemical Science* **2021**, *12* (14), 5185-5195. DOI: 10.1039/D0SC05707J.

(3) Hanozin, E.; Harper, C. C.; McPartlan, M. S.; Williams, E. R. Dynamics of Rayleigh Fission Processes in ~100 nm Charged Aqueous Nanodrops. *ACS Central Science* **2023**, *9* (8), 1611-1622. DOI: 10.1021/acscentsci.3c00323.

(4) McPartlan, M. S.; Harper, C. C.; Hanozin, E.; Williams, E. R. Ion emission from 1–10 MDa salt clusters: individual charge state resolution with charge detection mass spectrometry. *Analyst* **2024**, *149* (3), 735-744. DOI: 10.1039/D3AN01913F.
